# Development and evaluation of an intervention to improve food and nutrition literacy among Iranian Kurdish primary school children: An application of intervention mapping approach

**DOI:** 10.3389/fpubh.2022.1059677

**Published:** 2023-01-04

**Authors:** Mohammad Ahmadpour, Nasrin Omidvar, Elham Shakibazadeh, Azam Doustmohammadian, Abbas Rahimiforoushani

**Affiliations:** ^1^Department of Health Education and Promotion, School of Public Health, Maragheh University of Medical Sciences, Maragheh, Iran; ^2^Department of Community Nutrition, Faculty of Nutrition Sciences and Food Technology, National Nutrition and Food Technology Research Institute, Shahid Beheshti University of Medical Sciences, Tehran, Iran; ^3^Department of Health Education and Promotion, School of Public Health, Tehran University of Medical Sciences, Tehran, Iran; ^4^Gastrointestinal and Liver Diseases Research Center, Iran University of Medical Sciences, Tehran, Iran; ^5^Department of Epidemiology and Biostatistics, School of Public Health, Tehran University of Medical Sciences, Tehran, Iran

**Keywords:** intervention, food and nutrition literacy, Iran, Kurdish, primary children, intervention mapping

## Abstract

**Background:**

Food and nutrition literacy (FNLIT) is a relatively new term that is used to define the knowledge, skills, and behaviors necessary to achieve a healthy diet. Improving food and nutritional literacy in children is a necessary solution to eliminate nutritional disorders in this age group. The purpose of this study was to design, implement and evaluate an intervention to improve food and nutrition literacy in children aged 10–12 years old based on an intervention mapping (IM) approach.

**Methods:**

This experimental study was performed in three phases. Through the first phase, an intervention was developed using the (IM) approach. In the second phase, the intervention was implemented for 6 months, and in the third phase, the intervention outcomes were evaluated and compared with the control group through a randomized controlled trial among 300 participants (each control and intervention group = 150).

**Results:**

Before the intervention, there was no significant difference between the control and intervention groups in all subscales of FNLIT. After the intervention, there was a significant difference between the control and intervention groups in all subscales of FNLIT (*P* < 0/001). There were no differences between the two groups in terms of the FNLIT scores at baseline (*P* > 0.05). However, after 6 months of intervention, a significant difference was observed between the two groups (intra-group differences) (*P* < 0.001). Such a difference was not identified in the control group after 6 months also the results show the impact of socioeconomic factors and parental literacy on the average score of students' FNLIT and after the intervention, a significant difference was observed between the scores of control and intervention groups in all subscales of the FNLIT (*P* < 0.001).

**Conclusions:**

The school-based intervention developed and evaluated in this study provides a basis for future programs targeting the improvement of FNLIT in children, especially in poor and deprived areas such as Kurdistan province.

**Clinical trial registration:**

Iranian Clinical Trials Registry (IRCT) Code: 32094.

## 1. Introduction

According to the latest World Health Organization report, non-communicable diseases are responsible for 36 million deaths annually, close to 80% (29 million people) of which occurs in low and middle-income countries (1). The main risk factors for chronic diseases are unhealthy nutrition, inadequate physical activity, and tobacco use (2). These risk factors often develop during childhood and adolescence and become institutionalized until adulthood (3). Among the four main risk factors of non-communicable diseases (NCDs), the diet has a greater role than the combination of physical activity, smoking, and alcohol consumption (4). Nutrition and diet have a major impact on population health and are associated with premature death and disability-adjusted life years (DALYs). The many years of life lost, poor quality of life, and NCDs impose an additional economic burden on society at high costs (5). Many risk factors for cancer and obesity worldwide are somehow linked to nutrition and diet (6).

Urbanization and its related lifestyle have resulted in major changes in the quantity and quality of nutritional patterns and physical activity, especially in children and adolescents (7, 8). In many parts of the world, these age groups supply about 50% of their calories from unhealthy food sources, highly processed foods, and drinks (9). Evidence in Iran also indicates the role of high-risk dietary behaviors in the prevalence of various nutritional problems, including malnutrition and communicable diseases (10, 11).

The basic point is that most of these health problems can be prevented through regular implementation of health promotion programs, including promoting healthy behaviors and changing lifestyles from early childhood (12). Childhood and adolescence are important periods for acquiring knowledge and skills related to food and nutrition (13).

Food and nutrition literacy (FNLIT) is a relatively new and multidimensional concept that may play an important role in establishing the overall dietary framework. It comprises a combination of necessary knowledge, skills, and practices relevant to nutritional recommendations (14–16), as well as a set of social, cultural, and ethnic factors (17, 18).

Despite conclusive evidence on the link between food literacy and diet quality (18–21), food literacy/nutrition literacy research focused on children is scarce (19, 20). Attention to FNLIT is very important in elementary school children because it is the starting point for food-related behaviors and skills education (21–23) and can empower them to choose healthy foods and adopt healthy eating behaviors throughout their lives (24, 25).

In Iran, interventions in nutrition education to enhance the nutritional knowledge of different age groups indicate the importance of paying attention to nutrition education. However, all aspects of FNLIT in children have not been provided formally and executively (18, 26–28). Given the effect of long-term contact with children at an early age and the interdependence between health and learning, schools are one of the most appropriate and important settings for preventive health promotion programs. School-based interventions provide access to young children early when unhealthy eating behavior has not yet begun (29, 30). Therefore, the present study aimed to design and evaluate an intervention to improve FNLIT in Iranian Kurdish primary school children using the IM approach. Intervention mapping (IM) is a process and unique approach for developing theory and evidence-based health education programs. Different studies have been performed to solve health problems using an interventional mapping approach, including a self-care training program for type 2 diabetic patients (31), designing educational interventions to promote food and nutrition literacy in primary school children (32), designing, implementing, and evaluation of community-based and school-based multifaceted intervention to sustainably increase fruit and vegetable consumption among children (33). The implementation of IM as a roadmap for designing and planning programs to address health problems has attracted the attention of researchers and planners in recent years (34).

## 2. Methods

### 2.1. Study design

This experimental field trial is part of a comprehensive study with three phases of intervention development, implementation, and evaluation. The study design was based on the IM approach. First introduced in 1998 by Bartholomew et al., IM comprises six steps, as follows: (1) needs assessment, (2) creating change goals matrix, (3) theory-based operational solutions, (4) program design, (5) program acceptance and implementation, and (6) program evaluation (35). Within the three phases of the study, four sub-studies were planned, comprising the six steps of the IM approach:

Phase 1, needs assessment and the first step of the IM, consisted of three sub-studies. In sub-study 1, the FNLIT questionnaire previously designed by Doustmohammadian et al. (18) for Tehran students was adapted for the Kurd-speaking subjects. Schools in Bane city, a city in the Kurdistan province, were chosen as the study setting. In sub-study 2, through a cross-sectional study, FNLIT status in a sample of 390 elementary students (grades 4–6) was assessed using the adapted questionnaire. In sub-study 3, through a qualitative phenomenological study, barriers and facilitators, the most important and practical measures, and the most appropriate channel(s) and intervention methods to promote FNLIT in the targeted population were identified. In the second phase, using the results obtained from the first phase studies steps 2–6 of the IM were implemented, leading to the development of an intervention. In the third phase, the effect of the designed intervention programs *via* a randomized controlled trial on 300 elementary school students aged 10–12 years in grades 4–6 (150 controls and 150 in the intervention group) was evaluated. Based on the needs assessment and resource review results, interventions to promote food and nutrition literacy in students were conducted in three sections, namely curriculum development, intervention implementation, and intervention evaluation, respectively.

**Figure 1:**
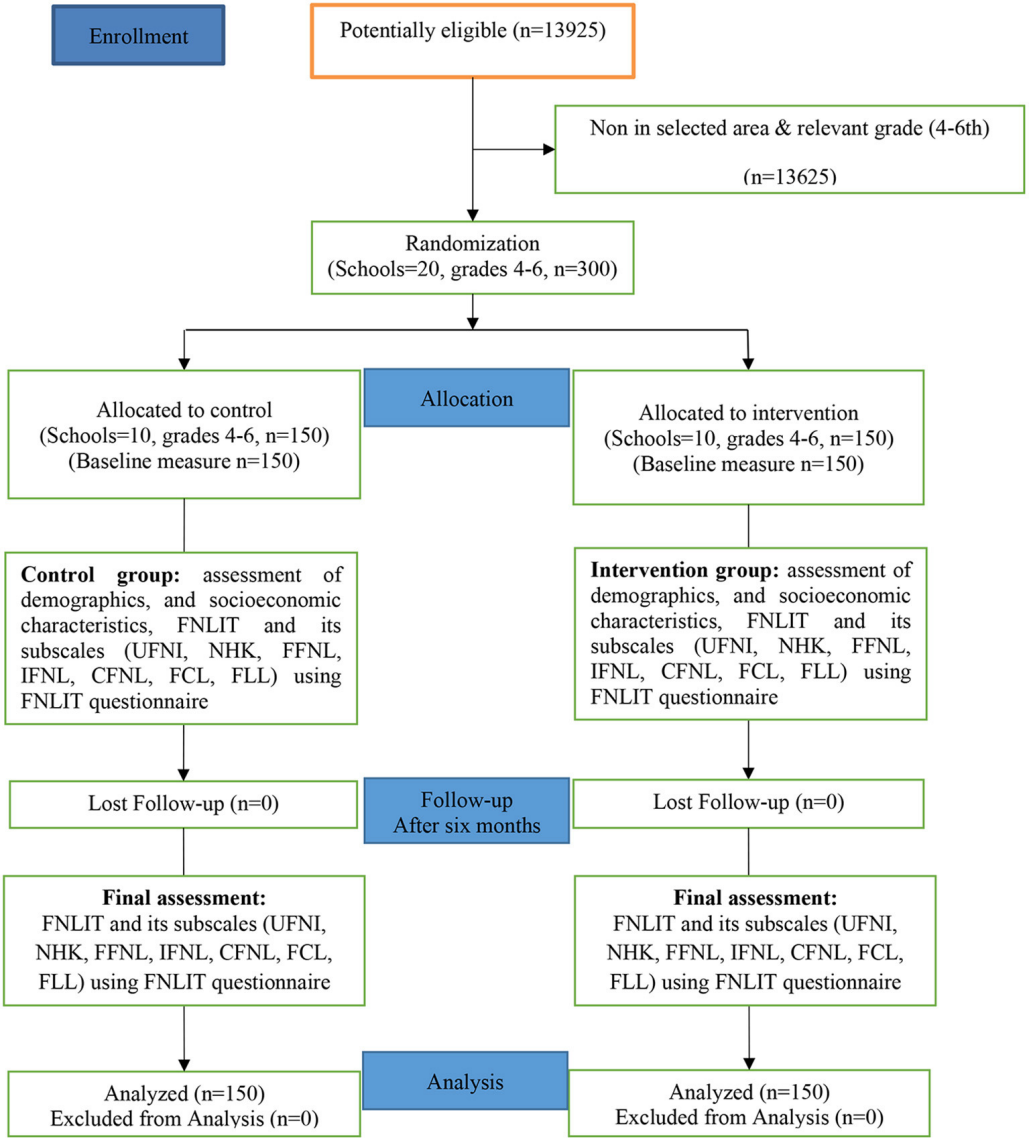
CONSORT flow diagram.

The study followed the CONSORT guideline for the design and reporting of clinical trials (S1 CONSORT Checklist), and the CONSORT flow diagram for the study is shown in Figure 1.

### 2.2. Sampling

Participants were fourth- to sixth-graders (10–12-year-olds) selected through stratified random sampling. The sample size was calculated considering the change in the mean and standard deviation of nutritional literacy scores based on the previous studies (36) using the following equation (37):


$$N\;=\;\frac{2\;{\left(Z_{\alpha }+\;Z_{\beta }\right)}^{2}\;(1+\left(n-1\right)\rho )}{n{\left[(\mu_{1}-\mu_{2})/\sigma \;\right]}^{2}}$$

Significance level = 0.05, Study power = 80%, Number of time repeats = 2.

Effect size: 0.179 based on the study by Appleton et al. (36). Correlation between observations = 0.5.

Based on the above calculations, the sample size in each group was calculated at 131 individuals. A total of 300 participants (150 students for intervention and 150 controls) were selected to account for possible sample loss. The inclusion criteria were students' desire to participate in research, family's desire to cooperate in the study, study in the fourth, fifth and sixth grades of elementary school, having no chronic illness, having no physical disability, Iranian citizenship, and not participating in the sub-study 2.

### 2.3. Data collection and participants

After the random allocation of schools to two groups of intervention and control with similar characteristics, equal numbers of girls and boys schools were assigned to the intervention and control groups. Thus, a total of 20 schools (10 as controls and 10 as interventions) were randomly selected, four from each geographical district (north, south, east, west, and downtown) of Baneh city. Before the commencement of the study, coordination with the management of the city's education department and all school administrators was achieved through a written letter and attendance at schools. All 4, 5, and 6 graders were selected in each school. Then, the benefits of participating in the study, how the program was implemented, and how long it took were explained to the school authorities, students, and parents, and the written informed consent form was completed and signed by the students and their parents.

Further explanation is provided in the intervention method section. At the beginning of the study (October 2018), data on demographics and socioeconomic status, FNLIT subscales, including the understanding of food and nutrition information (UFNI), nutritional health knowledge (NHK), functional food and nutrition literacy (FFNL), interactive food and nutrition literacy (IFNL), food choice literacy (FCL), critical food and nutrition literacy (CFNL), food label literacy (FLL) were collected from students in both intervention and control groups by a questionnaire. After 6 months of implementation of the education program (March 2019), students in both control and intervention groups completed the same questionnaire.

Due to the nature of the treatment, it was impossible to blind subjects to educational and environmental interventions. The project coordinator and research assistants were also not blinded because they were responsible for implementing the intervention.

### 2.4. Curriculum

Using the results obtained from phases 1 and 2, which included designing steps 2–6 of the IM and considering the expected changes in knowledge, skills, and behaviors of the main and sub-target groups, the educational program content was designed. The final curriculums for children, parents, and school staff are presented in Table 1.

**Table 1:** Educational curriculum for children, parents, and school staff.

**Participants**	**Cognitive and behavioral change goals**	**Time and number of training sessions/** **practical work**	**Trainer**	**Teaching/learning strategies**	**Training session titles**
Children (10–12 y)	Promotion of the: 1. Understanding of food and nutrition information 2. Nutritional health knowledge 3. Functional food and nutrition literacy 4. Interactive food and nutrition literacy 5. Food choice literacy 6. Critical food and nutrition literacy 7. Food label literacy	16 forty-five minute sessions	Nutritionist/Teacher	1. Lecture 2. Group discussion 3. Questions and answers 4. Brain storm 5. Demo games 6. Practical work 7. Field trip and scientific tour	1.Know the main food groups and what is their nutritional value Bread and cereals group from farm to the tablecloth 3. Fruits and vegetable group 4. Let's make a healthy Snack meal together 5. Let's get acquainted Milk & Dairy group 6. Meat, bean, and kernel nutrients: important sources of protein 7. Get to know healthy drinks 8. How to read and understand a nutritional label on a food product? 9. Learning to read labels in food stores and shopping malls 10. Do food ads give us the right information? 11. Let's review a food ad 12. How to say “No” to unhealthy eating offerings? 13. How do we get answers to our food questions? 14. How to help my parents choose healthy foods when shopping? 15. How can we make healthy food choices with what we've learned so far? 16. Browsing the topics we've learned so far
Parents	1. Encouraging children to prepare their own snacks 2. Help children learn to say “no” to unhealthy food	3–5 ninety minute sessions 3. Help children learn the right nutrition choices 4. Remove unhealthy foods from the household food basket with the help of children	Nutritionist		
**School staff	1. Encourage students to discuss and share food and nutrition information with peers and parents 2. Assist students in analyzing and criticizing food and nutrition advertising in the media 3. Assist students in analyzing and criticizing nutrition labels 4. Helping students adhere to healthy eating patterns in the school environment 5. Prevent the distribution and consumption of unhealthy food in the school environment	3–5 ninety-minute sessions	Nutritionist/School counseling expert		

### 2.5. Intervention

The designed intervention included two components: 1) nutrition education and 2) environmental interventions.

#### 2.5.1. Educational interventions

The education program targeted two groups: (1) parents and school staff and (2) students (main target).

Nutrition experts conducted parent and school staff training in three curriculum-based training sessions. For each session, a school health expert was appointed as the supervisor to oversee the sessions and provide a written report to the project manager at the end of the session. Three educational worksheets were prepared for each training session. Through these assignments that were supposed to be followed over 1 month, parents were obliged to follow the instructions as requested, and then the results were reviewed at the end of each month, and feedback was given to the parents.

Student training was planned in 16 sessions according to the educational curriculum for a period of 16 weeks in the fall and winter of the academic year 2018–2019. The sessions were held by teachers and supervised by a nutrition expert in the school classrooms. Teaching/learning strategies included the followings: (1) lecture, (2) group discussions, (3) questions and answers, (4) brainstorming, (5) demo games, (6) practical work, and (7) field trip and scientific tour. One week prior to each training session, coordination was made with the teacher and school staff, and all team members were informed of any possible changes.

#### 2.5.2. Environmental interventions

The environmental interventions included activities aimed at creating a healthy food environment both at home and school. The goals of home interventions included: (1) increasing students' access to healthy food and limiting unhealthy food access at home; (2) increasing students' involvement in food shopping, preparation, and cooking at home, (3) involving students in home food decisions, (4) involving students in planting vegetables at home parents. The school environment intervention targeted the socio-cultural, physical, economic, and political environment, as follows: (1) modifying the school's Socio-Cultural environment through implementing simple and healthy food competitions, food and nutrition festivals, healthy nutrition painting contests among children; (2) improving the school's physical environment through distributing healthy snacks and hygienic foodstuffs at the school canteen; (3) modifying the school's economic environment by distributing healthy school meals at an affordable price or partially free-of-charge in the canteen; and (4) improving the school's political environment by initiating a School Food and Nutrition Committee composed of teachers, school food service staff, school health personnel, and delegates from students, parents, and other stakeholders to coordinate and support promotion activities, such as: installing banners, posters, and flyers to promote FNLIT, and changing school policies regarding food and nutrition education, as well as the type and characteristics of nutritional snacks available at the school store (Table 2).

**Table 2:** Environmental changes and the implementation strategies at home and school.

**Target setting**	**Change objectives**	**Strategies**
Home	1. Increasing students' access to healthy food and limiting unhealthy food access at home	Advocacy with parents
	2. Increasing students' involvement in food shopping, preparation, and cooking at home	
	3. Involving students in home food decisions.	
	4. Involve students in planting vegetables at home by parents	
School	1. Socio-cultural environment: organizing simple and healthy food competitions, food and nutrition festivals, and painting competitions on healthy nutrition	Advocacy and lobbying with the school officials
	2. Physical environment: distributing healthy nutrition snacks in the school buffet	
	3. Economic environment: distributing healthy nutrition at a properly controlled price at the school store	
	4. Political environment: forming a school food and nutrition committee consisting of teachers, school food service staff, school health personnel, and students to model these people and supporting food and nutrition promotion activities, banner installation, posters, and other environmental measures related to promoting food and nutrition literacy	

### 2.6. Process evaluation

During the implementation phase, in order to refine and improve educational sessions and their final effect, teaching methods and quality of the trainers, the degree of students' participation in the discussions, the ability to understand the content, the participant's satisfaction with the number, time and place of the training sessions, innovation in the content and among students, parents, and school staff were assessed by a checklist. Study participants were asked to indicate their final evaluation and satisfaction regarding the school and home environment in terms of healthy eating and nutrition before and after the program using a checklist.

### 2.7. Outcome evaluation

The main outcome measure of this intervention study was students' FNLIT scores. Using the FNLIT questionnaire, students' scores before and after intervention regarding different subscales, including understanding of food and nutrition information, nutritional health knowledge, functional food and nutrition literacy, interactive food and nutrition literacy, food choice literacy, critical food and nutrition literacy, food label literacy and total food and nutrition literacy were compared.

### 2.8. Data analysis

The normality of the data was evaluated by the Kolmogorov-Smirnov test. Since the data distribution in both control and intervention groups was not normal, we used the Wilcoxon, Man-Whitney U, and Kruskal-Wallis H statistical tests for data analysis. In order to compare the FNLIT subscales before and after the intervention within and between groups, Wilcoxon and Mann-Whitney U tests were used, respectively. Wilcoxon and Mann-Whitney U tests were used to determine the relationship between FNLIT status with demographic variables in dichotomous variables such as gender before and after the intervention within and between groups. Wilcoxon and Kruskal-Wallis H tests were used in multi-state variables such as the number of family members before and after the intervention within and between groups.

## 3. Results

The demographic and social characteristics of the participants are shown in Table 3. There were no differences between the two groups regarding the FNLIT scores at baseline (*P* > 0.05). However, after 6 months of intervention, a significant difference was observed between the two groups (Intra-group differences) (*P* < 0.001). Such a difference was not identified in the control group after 6 months.

**Table 3:** Demographic and social characteristics of student participants (*n* = 300).

**Variable**	**Sub-group**	**Total (*n* = 300)**	**Intervention (*n* = 150)**	**Control (*n* = 150)**
		***n*** **(%)**	***n*** **(%)**	***n*** **(%)**
**Child characteristics**				
Age	11 ± 1 y	100 (100)	150 (50)	150 (50)
Sex	Female	150 (50)	75 (25)	75 (25)
	Male	150 (50)	75 (25)	75 (25)
Birth Order	1	121 (40.3)	68 (22.6)	53 (17.7)
	2	90 (30)	41 (13.7)	(16.3) 49
	3	46 (15.3)	21 (7)	25 (8.3)
	4 and more	43 (14.3)	20 (6.6)	23 (7.7)
**Household characteristics**				
Family size	3	78 (25.9)	44 (14.6)	34 (11.3)
	4	135 (45.1)	65 (21.7)	70 (23.4)
	5	43 (14.3)	20 (6.6)	23 (7.7)
	6 and more	44 (14.7)	21 (7)	23(7.7)
Father's age	< 35y	76 (25.3)	38 (12.7)	38 (12.7)
	35–44 years	149 (49.7)	71 (23.7)	78 (26)
	>44 years	75 (25)	41 (13.6)	34 (11.4)
Mother's age	< 35 years	65 (21.8)	28 (9.4)	37 (12.4)
	35–44 years	148 (49.2)	76 (25.2)	72 (24)
	>44 years	87 (29)	46 (15.3)	41 (13.7)
Father's education level	Illiterate	31 (10.3)	16 (5.3)	15 (5)
	Elementary	111 (36.7)	55 (18.3)	56 (18.4)
	3rd middle school	70 (23.5)	40 (13.5)	30 (10)
	Diploma	58 (19.5)	24 (8)	34 (11.5)
	Associate degree and above	30 (10)	15 (5)	15 (5)
Mother's education level	Illiterate	51 (17.1)	29 (9.7)	22 (7.4)
	Elementary	150 (50)	70 (23.4)	80 (26.7)
	3rd middle school	64 (21.4)	34 (11.4)	30 (10)
	Diploma	26 (8.6)	14 (4.6)	12 (4)
	Associate degree and above	9 (3)	3 (1)	6 (2)
Father's occupation	Unemployed	37 (12.3)	17 (5.6)	20 (6.7)
	Employee	67 (22.3)	33 (11)	34 (11.3)
	Worker	196 (65.3)	100 (33.3)	96 (32)
Mother's occupation	Employed	284 (94.6)	141 (47)	143 (47.6)
	Housewife	16 (5.3)	9 (3)	7 (2.4)

Since more than 98% of the participants were Kurds and spoke Kurdish, ethnicity and language were excluded from the list of confounding variables for FNLIT in the analysis. Age of half of the parents was 44–35 years (control = 52% and intervention = 47.4%). Most participants were the first children in the family (control = 35.4% and intervention = 45.2%). The four-person families had the highest number in both groups (control = 46.8% and intervention = 43.4%). Moreover, the parental education level of most participants was at the elementary level, and based on the results obtained from the Kruskal Wallis test listed in Table 4, it shows the impact of socioeconomic factors and parental literacy on the average score of students' food and nutrition literacy.

**Table 4:** Comparison of FNLIT scores based on the demographic and social characteristics in both groups before and after the intervention.

**Characteristic**			**Control (*n* = 150) Mean ±(SD)**	**Intervention (*n* = 150) Mean ±(SD)**	**Kruskal-Wallis test result**		
					**Chi. S. value**	**df**	*P* ^***^
Age (year)	10	Before	57.1 (5.8)	58.6 (5.8)	1.440	1	0.230
		After	57.2 (6.3)	95.1 (7.7)	75.601	1	< 0.001
		P^**^(Z)	0.943 (−0.071)	< 0.001 (−6.15)			
	11	Before	59.8 (6.3)	60.5 (6.4)	0.305	1	0.580
		After	59.1 (6.6)	95.4 (7.7)	75.5	1	< 0.001
		P^**^(Z)	0.726 (−0.35)	< 0.001 (−6.15)			
	12	Before	58 (5.5)	56.6 (5.3)	1.292	1	0.256
		After	57.3 (5.2)	95.9 (6.7)	75.508	1	< 0.001
		P^**^(Z)	0.148 (−1.446)	< 0.001 (−6.15)			
Sex	Female	Before	59 (5.8)	59.4 (6.4)	*P* ^*^		Z
					0.776		−0.284
		After	58.8 (5.7)	95.2 (7.9)	< 0.001		−10.675
		P^**^(Z)	0.629 (−0.483)	< 0.001 (−7.52)	*P* ^*^		Z
	Male	Before	57.5 (6)	57.8 (5.6)	−0.749		−0.320
		After	56.8 (6.3)	95.8 (6.8)	< 0.001		−10.650
					Chi.S. Value	df	*P* ^***^
		P^**^(Z)	0.604 (−0.518)	< 0.001 (−7.52)			
Birth order	1	Before	56.8 (5.3)	57.7 (5.8)	0.141	1	0.707
		After	56.5 (5.4)	95.8 (7.5)	92.09	1	< 0.001
		P^**^(Z)	0.758 (−0.308)	< 0.001 (−7.11)			
	2	Before	58.2 (5.9)	58.9 (5.9)	0.416	1	0.519
		After	58.1 (5.8)	94.9 (7.1)	66.68	1	< 0.001
		P^**^(Z)	0.390 (−0.859)	< 0.001 (−5.57)			
	3	Before	59.3 (6.2)	57.9 (5.4)	0.634	1	0.426
		After	58.4 (6.8)	95.9 (6.8)	33.98	1	< 0.001
		P^**^(Z)	0.123 (−1.542)	< 0.001 (−4.01)			
	4 and more	Before	60.6 (6.7)	61.3 (7.2)	0.274	1	0.601
		After	59.7 (7.1)	95.3 (8)	31.91	1	< 0.001
		P^**^(Z)	0.401 (−0.840)	< 0.001 (−4.01)			
Father's age	< 35 years	Before	58.1 (4.9)	59.8 (6.4)	1.001	1	0.317
		After	57.5 (5)	96.5 (7)	57.774	1	< 0.001
		P^**^(Z)	0.871 (−0.162)	< 0.001 (−5.44)			
	35–44 years	Before	58.1 (6.1)	57 (5.7)	1.282	1	0.257
		After	57.3 (6.3)	96.4 (6.3)	112.989	1	< 0.001
		P^**^(Z)	0.063 (−1.862)	< 0.001 (−7.32)			
	>44 years	Before	58.8 (6.7)	60 (5.7)	0.838	1	0.361
		After	59.3 (6.6)	92.9 (8.8)	55.617	1	< 0.001
		P^**^(Z)	0.148 (−1.448)	< 0.001 (−5.51)			
Mother's age	< 35 years	Before	58.2 (5.8)	59.5 (6)	0.763	1	0.382
		After	57.2 (6.3)	95.3 (8)	47.881	1	< 0.001
		P^**^ (Z)	0.162 (−1.397)	< 0.001 (−4.703)			
	35–44 years	Before	58.3 (6.1)	58.1 (6.2)	0.065	1	0.798
		After	57.8 (6)	96.5 (6.3)	113.03	1	< 0.001
		P^**^ (Z)	0.600 (−0.525)	< 0.001 (−7.576)			
	>44 years	Before	58.2 (6)	58.7 (5.8)	0.222	1	0.637
		After	58.4 (6.2)	93.9 (8.2)	65.125	1	< 0.001
		P^**^ (Z)	0.230 (−1.201)	< 0.001 (−5.841)			
Father's education	Illiterate	Before	59.4 (5.6)	60.7 (5.8)	0.230	1	0.566
		After	58.4 (5.6)	93.3 (9.8)	23.323	1	< 0.001
		P^**^ (Z)	0.593 (−0.535)	< 0.001 (−3.517)			
	Elementary	Before	58.7 (5.5)	57.5 (5.9)	1.587	1	0.208
		After	58.3 (5.3)	94.4 (6.3)	83.879	1	< 0.001
		P^**^ (Z)	0.258 (−1.130)	< 0.001 (−6.452)			
	3rd middle school	Before	57.4 (6.8)	58.5 (5.7)	1.136	1	0.286
		After	57.5 (6.7)	95 (7.8)	52.236	1	< 0.001
		P^**^ (Z)	0.670 (−0.426)	< 0.001 (−5.444)			
	Diploma	Before	56.5 (5.4)	58.1 (6.1)	0.455	1	0.50
		After	56 (5.7)	95 (7.5)	41.817	1	< 0.001
		P^**^ (Z)	0.938 (−0.078)	< 0.001 (−4.288)			
	Associate technician and above	Before	61.2 (6.4)	61 (7)	0.002	1	0.967
		After	60.2 (8.3)	96.6 (6.8)	21.814	1	< 0.001
		P^**^ (Z)	0.735 (−0.339)	< 0.001 (−3.517)			
Mother's Education	Illiterate	Before	58.1 (5.1)	59.7 (5.3)	0.523	1	0.470
		After	58.6 (5.5)	88.8 (10.4)	37.129	1	< 0.001
		P^**^ (Z)	0.050 (−1.958)	< 0.001 (−4.703)			
	Elementary	Before	59.7 (5.8)	60.8 (5.4)	1.471	1	0.225
		After	59 (6)	97 (5.5)	113.371	1	< 0.001
		P^**^ (Z)	0.092 (−1.683)	< 0.001 (−7.220)			
	3rd middle school	Before	52.4 (2.6)	51.7 (2)	1.107	1	0.293
		After	52.3 (3.3)	97.6 (4.3)	48.476	1	< 0.001
		P^**^ (Z)	0.959 (0.052)	< 0.001 (−5.130)			
	Diploma	Before	58.6 (1)	58.3 (0.0)	1.167	1	0.280
		After	58.8 (1.8)	97.8 (1.6)	19.158	1	< 0.001
		P^**^ (Z)	0.50 (−0.674)	< 0.001 (−3.329)			
	Associate technician and above	Before	69.8 (0.0)	70.7 (1.7)	1.250	1	0.264
		After	65.7 (9.1)	92 (13.5)	5.554	1	0.018
		P^**^ (Z)	0.258 (−1.069)	0.066 (−1.841)			
Father's occupation	Unemployed	Before	57.2 (5.3)	58 (5)	0.112	1	0.738
		After	56.6 (5.6)	95.5 (7.6)	27.403	1	< 0.001
		P^**^ (Z)	0.248 (−1.156)	< 0.001 (−3.724)			
	Employee	Before	59.2 (5.4)	58.6 (6.1)	0.335	1	0.563
		After	58.1 (5.4)	95.7 (6.9)	49.998	1	< 0.001
		P^**^ (Z)	0.255 (−1.138)	< 0.001 (−5.013)			
	Worker	Before	58.2 (6.3)	58.6 (6.3)	0.438	1	0.508
		After	58 (6.4)	95.4 (7.5)	149.258	1	< 0.001
		P^**^ (Z)	0.739 (−0.334)	< 0.001 (−8.639)			
Mother's occupation	Employed	Before	58.1 (6)	58.5 (6)	*P* ^*^		Z
					0.630		−0.481
		After	57.7 (6.1)	95.4 (7.2)	< 0.001		−14.677
		P^**^ (Z)	0.694 (−0.393)	< 0.001 (−10.266)	*P* ^*^		Z
	Housewife	Before	62.2 (4.5)	59.8 (7.4)	0.478		−0.710
		After	60.8 (5)	96.5 (8.7)	< 0.001		−3.397
		P^**^ (Z)	0.059 (−1.89)	0.005 (−2.803)			
					Chi.S.	df	*P* ^***^
					Value		
Number of family members	3	Before	57.3 (5)	57.7 (5.6)	0.026	1	0.872
		After	57.1 (5.1)	95.8 (7.6)	59.141	1	< 0.001
		P^**^ (Z)	0.778 (−0.282)	< 0.001 (−5.712)			
	4	Before	57.9 (6)	58.4 (6)	0.257	1	0.612
		After	57.6 (6)	95.3 (7.2)	101.553	1	< 0.001
		P^**^ (Z)	0.888 (−0.141)	< 0.001 (−7.010)			
	5	Before	59.2 (5.9)	57.9 (5.6)	0.610	1	0.435
		After	58.3 (6.5)	96.7 (5.9)	31.901	1	< 0.001
		P^**^ (Z)	0.195 (−1.297)	< 0.001 (−3.924)			
	6 and more	Before	60.1 (6.9)	61.2 (7)	0.305	1	0.580
		After	59.1 (7.4)	94.6 (8.4)	32.663	1	< 0.001
		P^**^ (Z)	0.314 (−1.007)	< 0.001 (−4.108)			

P^*^ = Man-Whitney U Test, P^**^(Z)= Wilcoxon Test , P^***^ = Kruskal-Wallis H Test.

The number of subjects studied in both control and intervention groups was equal and they were identical in terms of age, gender, and educational levels in order to eliminate the confounding effect. Based on the results, the birth rank of one has the highest frequency in both control (35.3%) and intervention (45.3%) groups. Family size of four in both control (46.7%) and intervention (43.3%) groups were more frequent than others. The parents' age of most of the participants in both the control and intervention groups was between 35 and 44 years. The parent's education of most of the participants in both the control and intervention groups was the elementary level.

In both the control and intervention groups, the fathers of the study subject were mostly workers, and the mothers were housewives. In both the control and intervention groups, the breadwinner of the family were their father. Most of the participants in the study in both groups did not have support resources. In terms of housing ownership status, most of the study participants in both the control and intervention groups stated that they owned their housing.

At baseline, the FNLIT scores were not significantly different between the control and intervention groups (58.6 in the intervention and 58.3 in the control groups) (*P* = 0.75). However, after the intervention, a significant difference was observed between the scores of control and intervention groups in all subscales of the FNLIT (*P* < 0.001) (inter-group difference). In the intervention group, the highest and the lowest scores were related to the “food choice literacy (FCL)” (99.1) and “food label literacy (FLL)” (86.3) subscales, respectively.

After the intervention, the total FNLIT score was 57.8 in the control group compared to 95.5 in the intervention group (*P* < 0.001). The mean score of FNLIT in the control group before and after the intervention was not significantly different in all subscales, except for the “FLL” subscale (no intra-group difference), which significantly decreased in the control group (*P* = 0.009) (Table 5).

**Table 5:** Mean and standard deviation scores of FNLIT subscales in both control and intervention groups.

**FNLIT subscale**		**Control (*n* = 150)**	**Intervention (*n* = 150)**	***P*** ^*^(Z)
		**Mean ±(SD)**	**Mean ±(SD)**	
Understanding of food and nutrition information	Before	63.7 (10.4)	65.5 (10.6)	0.124 (−1.54)
	After	63.1 (11.1)	98.3 (3.1)	< 0.001 (−14.98)
P^**^(Z)	0.925 (-0.09)	< 0.001 (−10.65)		
Nutritional health knowledge	Before	81.5 (10.3)	82 (9.1)	0.994 (−0.008)
	After	80.4 (9.9)	97.5 (3.8)	< 0.001 (−14.05)
P^**^(Z)	0.057 (-1.9)	< 0.001 (−10.47)		
Functional food and nutrition literacy	Before	57.1 (13.3)	58.4 (14.3)	0.630 (−0.48)
	After	57.4 (13.3)	98.1 (3.7)	< 0.001 (−15.08)
P^**^(Z)	0.065 (-1.84)	< 0.001 (−10.63)		
Interactive food and nutrition literacy	Before	53.2 (10.4)	54 (10.3)	0.614 (−0.5)
	After	53.3 (10.5)	95.8 (8.5)	< 0.001 (−14.83)
P^**^(Z)	0.506 (-0.66)	< 0.001 (−10/6)		
Food choice literacy	Before	70.3 (11.7)	70.6 (11.9)	0.747 (−0.32)
	After	69.9 (11.5)	99.1 (2.2)	< 0.001 (−15.39)
P^**^(Z)	0.92 (-0.1)	< 0.001 (−10.59)		
Critical food and nutrition literacy	Before	54.5 (13.2)	52.7 (12.2)	0.369 (−0.898)
	After	54.9 (13.1)	93.4 (13)	< 0.001 (−13.96)
P^**^(Z)	0.291 (-1.05)	< 0.001 (−10.44)		
Food label literacy	Before	27.6 (9.3)	26.8 (6.5)	0.337 (−0.96)
	After	25.7 (9.1)	86.3 (21.5)	< 0.001 (−14.73)
P^**^(Z)	0.009 (-2.63)	< 0.001 (−10.64)		
Total food and nutrition	Before	58.3 (6)	58.6 (6)	0.750 (−0.31)
	After	57.8 (6.1)	95.5 (7.3)	< 0.001 (−14.85)
P^**^(Z)	0.688(-0.4)	< 0.001 (−10.62)		

P^*^(Z) = Man-Whitney U Test, P^**^(Z) = Wilcoxon Test.

## 4. Discussion

Based on the findings, the intervention designed and implemented in elementary schools in Baneh resulted in a significant improvement in total food and nutrition literacy (FNLIT) score and almost all its subscales in the intervention group compared to the controls. The FNLIT scores in both groups were moderate or low in more subscales before the intervention, except for the two subscales of the cognitive domain: understanding of food and nutrition information and nutritional health knowledge. The scores of both groups were moderate to low in all other subscales, especially in interactive food and nutrition literacy and food label literacy subscales. In the intervention group, the highest and the lowest scores were related to the “food choice literacy” and “food label literacy” subscales, respectively. These results are consistent with those reported by Doustumohammadian et al. in Tehran (19), indicating moderate literacy scores in the cognitive domain but low scores in almost all skill subscales. Also, analysis of the content of primary school textbooks in Iran has shown that there is more emphasis on cognitive aspects of food and nutrition, with little emphasis on developing practical food and nutrition skills (38, 39). Similar results have been reported in other studies, and this finding reemphasizes the need to reform educational approaches to focus more on hands-on practice experiences, specifically when it comes to everyday life skills (40–42).

The present study indicates a considerable gap between FNLIT in the cognitive and skill domains in children aged 10–12 years. These findings reemphasize the need to reform educational approaches to focus more on hands-on practice experiences, specifically when it comes to everyday life skills (43–45). A comprehensive educational program should provide strategies to improve learners' knowledge, as well as skills needed to develop and practice intended behaviors, including healthy nutrition behaviors (39). Combining food and nutrition literacy encompasses a broad range of skills and abilities considered in food literacy (skill in food choice and preparation), as well as skills in nutrition literacy, such as how to access sound nutritional information and interaction and communication skills with others (46). Such skills can complement food skills and may improve the total impact. Therefore, the present study combines and utilizes both aspects of food literacy and nutrition literacy to cover the range of issues that needs to be addressed by both. Also, special attention was given to the community ethnic culture (47). Recent studies consider FNLIT rather than a one-dimensional view as an integrated structure encompassing various socioeconomic and ecological dimensions (43, 46, 48). Beatrice Velpini and others obtained almost heterogeneous results that showed different spectra. The effective types of food and nutrition literacy interventions included the following characteristics: technology components, involvement of different methods, time period of more than 1 month, and face-to-face meetings (44). Furthermore, West et al., in a before-and-after study that investigated the value of a 6-week program called OzHarvest NEST (Education and training of nutrition skills) in promoting food security and nutritional literacy, concluded that achieving food security requires multifaceted features and upstream determinants (45).

Using the IM approach was a strength of the present study. The advantage of using the intervention mapping approach has been demonstrated in various studies. A literature review suggests that the intervention mapping approach, with emphasis on evidence and theory, provides an appropriate conceptual framework for guiding the content and strategies used to design health promotion interventions, and interventions designed using this framework positively affect health outcomes. Krolner et al. (33), conducted a study aimed at designing, implementing, and evaluating a community-based and school-based multifaceted intervention to sustainably increase fruit and vegetable intake among 13-year-olds (seventh grade). This study used an interventional approach to guide the intervention design. The results showed that the approach taken had provided insights into effective strategies for increasing fruit and vegetable intake among adolescents. The systematic and theory-based approach used in this study can guide the design of process evaluations in future interventional studies and practices (33). Other studies that used IM to design and implement nutrition interventions have reported positive effects on improving short-term outcomes (22, 31). Based on such evidence, the present study applied the steps of the intervention mapping approach to design, implement and evaluate the intervention (17, 22, 31, 32).

In Iran, school food environments do not usually support students' healthy food choices. Often school staff and peers, as students' main role models, do not have appropriate eating patterns most of the time, and school stores generally do not adhere to healthy nutrition policies (49, 50). Therefore, it is necessary not only to reevaluate curriculums regarding FNLIT but also to reemphasize healthy food environment policies in Iranian schools. This approach will provide a supportive environment to back up what is being learned in the classroom.

Children have a strong interest in food and nutrition information and learning and practicing food and nutrition skills, and they usually welcome happy, varied, inexperienced, and multifaceted intervention programs, and they consider the school a good place to start these programs.

Based on the evidence, multi-level and multi-component interventions have often been successful (51–53). Several intervention studies in Iranian schools have previously aimed at teaching healthy eating topics (26–28); however, this is the first attempt to promote FNLIT in Iranian children.

## 5. Strengths and limitations

Although this study presents new findings on promoting FNLIT in primary children, it also has some limitations. The most important of these limitations are the following. First, the inconsistency of some unhealthy food advertising on national media with the nature of the study curriculum, that the effect of unhealthy food advertisements on ongoing and sustained training, group discussions, and criticism of unhealthy food advertising in the national media counteracted the target group.

Second, the lack of inter-sectoral cooperation between the Ministry of Health and the Ministry of Education to provide children with proper education on healthy nutrition and promote their food and nutrition literacy has exacerbated the dilemma and should be addressed at the national level by health policymakers. Third, children usually prefer unhealthy foods to natural and healthy ones because of their flavors/taste. Many children know healthy foods and may have access to them but still choose to eat unhealthy ones. In order to change this, the issue must be addressed in a multifaceted and inclusive way, and all the different sections involved should take part in resolving it. Finally, the generalizability of the results was limited to Iranian primary school students in the Baneh region in Kurdistan.

## 6. Conclusions

The results of the present study showed that the educational interventions designed and implemented could effectively promote FNLIT at the elementary school level, even in a deprived community, in terms of educational and nutrition facilities. This may indicate the value and importance of teamwork as well as active and practical classroom activities. Also, the active participation of the participants and systematic, ecologically-, fact-based planning of the intervention may have affected its results. Due to the achievements of the study and as a result of advocacy made by the schools' staff, it was approved by the Health Committee of the general director of education in Baneh to include the intervention in the curriculum of all elementary schools for the coming academic year.

## 7. Implications of research findings

This FNLIT promotion study, using the intervention mapping approach, involved students, parents, and school personnel and utilized an ecological perspective. We designed the intervention based on indigenous culture and utilized the native potentials and capabilities of the local community. This should be emphasized in future interventions to promote children's food and nutrition literacy. The extensive use of national and social media is also suggested to cover a large group of people.

The need to include food and nutrition literacy, including its analytical, interactive, and functional aspects, in the current school curricula is highly emphasized.

## Author's note

This is part of a Mixed Methods Study for a Ph.D. thesis of the first investigator (MA) at the Department of Health Education and Promotion, School of Public Health, Tehran University of Medical Sciences, Tehran, Iran.

## Data availability statement

The raw data supporting the conclusions of this article will be made available by the authors, without undue reservation.

## Ethics statement

The studies involving human participants were reviewed and approved by School of Public Health and Allied Medical Sciences, Tehran University of Medical Sciences. Written informed consent to participate in this study was provided by the participants' legal guardian/next of kin. Written informed consent was obtained from the minor(s)' legal guardian/next of kin for the publication of any potentially identifiable images or data included in this article.

## Author contributions

ES and NO were joint supervisors of the thesis. AR and AD were the study advisors. All authors contributed to all aspects of the investigation. All authors read and approved the final version.
