# Supplementary material for: Development and evaluation of an intervention to improve food and nutrition literacy among Iranian Kurdish primary school children: An application of intervention mapping approach
Source: Front Public Health. 2023 Jan 4;10:1059677. doi: 10.3389/fpubh.2022.1059677 (PMC9845926; doi:10.3389/fpubh.2022.1059677)
Supplement: Supplementary file 1 [file Data_Sheet_1.pdf]

Food and Nutrition Literacy (FNLIT) scale for elementary school children

Please carefully read each statement and check your response. Please check only one box for each statement.

	Statements
1.	**When shopping, how important is the nutritional information about food ingredients for you?**<br>☐Not important at all ☐Not very important ☐Neutral ☐Somewhat important ☐Very important
2.	**When shopping, how important is standardized labeling on food packages for you?**<br>☐Not important at all ☐Not very important ☐Neutral ☐Somewhat important ☐Very important
3.	**When shopping, how important are production and expiration dates for you?**<br>☐Not important at all ☐Not very important ☐Neutral ☐Somewhat important ☐Very important
4.	**I can easily understand the nutrition facts (e.g., amount of energy, sugar, protein, etc.) on food packages.**<br>☐Never ☐Seldom ☐Sometimes ☐Usually ☐Always
5.	**I can easily understand nutritional issues I read about in newspapers, magazines, and brochures.**<br>☐Never ☐Seldom ☐Sometimes ☐Usually ☐Always
6.	**I can understand nutritionists' recommendations about health and nutritional requirements that are appropriate for my age group.**<br>☐Never ☐Seldom ☐Sometimes ☐Usually ☐Always
7.	**Boiling is one of the more healthy cooking methods.**<br>☐Strongly agree ☐Agree ☐Neither agree nor disagree ☐Disagree ☐Strongly disagree
8.	**I can understand information and recommendations about proper nutrition for children in the media (e.g., TV, Internet, radio, etc.)**<br>☐Never ☐Seldom ☐Sometimes ☐Usually ☐Always
9.	**Daily physical activity for 30–40 minutes prevents obesity.**<br>☐Strongly agree ☐Agree ☐Neither agree nor disagree ☐Disagree ☐Strongly disagree
10.	**I know how different vegetables are cultivated and grown.**<br>☐Understand very well ☐Understand fairly well ☐Understand somewhat ☐Understand poorly ☐ Don't understand at all
11.	**Consumption of salty snacks (e.g., chips, corn puffs, etc.) is harmful for health.**<br>☐Strongly agree ☐Agree ☐Neither agree nor disagree ☐Disagree ☐Strongly disagree
12.	**Excessive consumption of sugar, sweets, and chocolate is harmful for health.**<br>☐Strongly agree ☐Agree ☐Neither agree nor disagree ☐Disagree ☐Strongly disagree
13.	**Consumption of salami and sausage that are high in fat may cause obesity.**<br>☐Strongly agree ☐Agree ☐Neither agree nor disagree ☐Disagree ☐Strongly disagree
14.	**Consumption of salami and sausage may cause cancer.**

	☐Strongly agree ☐Agree ☐Neither agree nor disagree ☐Disagree ☐Strongly disagree
**15.**	**Reading of production and expiration dated on food packaged is important for health.**<br>☐Strongly agree ☐Agree ☐Neither agree nor disagree ☐Disagree ☐Strongly disagree
**16.**	**I eat a variety of vegetables (e.g., lettuce, cabbage, tomatoes, carrots, etc.), every day.**<br>☐Never ☐Seldom ☐Sometimes ☐Usually ☐Always
**17.**	**I share the nutritional issues that I obtain from various sources with others (e.g., friends, family, etc.)**<br>☐Never ☐Seldom ☐Sometimes ☐Usually ☐Always
**18.**	**I talk to my friends and family about healthy eating.**<br>☐Never ☐Seldom ☐Sometimes ☐Usually ☐Always
**19.**	**If I have any questions about food and nutrition issues, I'm able to get information and advice from parents, teachers, etc.**<br>☐Never ☐Seldom ☐Sometimes ☐Usually ☐Always
**20.**	**I prepare my own snacks for school.**<br>☐Never ☐Seldom ☐Sometimes ☐Usually ☐Always
**21.**	**I bring healthy snacks to school.**<br>☐Never ☐Seldom ☐Sometimes ☐Usually ☐Always
**22.**	**I regularly do exercise or walk for 30 to 40 minutes every day.**<br>☐Never ☐Seldom ☐Sometimes ☐Usually ☐Always
**23.**	**I wash and prepare fruits and vegetables myself.**<br>☐Never ☐Seldom ☐Sometimes ☐Usually ☐Always
**24.**	**I eat fruit every day.**<br>☐Never ☐Seldom ☐Sometimes ☐Usually ☐Always
**25.**	**I eat breakfast every day.**<br>☐Never ☐Seldom ☐Sometimes ☐Usually ☐Always
**26.**	**I have enough will power to resist unhealthy foods (e.g., fast food, pizza, carbonated drinks, etc.)**<br>☐Never ☐Seldom ☐Sometimes ☐Usually ☐Always
**27.**	**If I go to restaurant or fast food with my friends, and all of them choose unhealthy foods (e.g., pizza, French fries, carbonated drinks, etc.), I'm able to choose healthy foods.**<br>☐Never ☐Seldom ☐Sometimes ☐Usually ☐Always
**28.**	**I can easily say "No" to any unhealthy eating suggestions from my friends.**<br>☐Never ☐Seldom ☐Sometimes ☐Usually ☐Always
**29.**	**If I encounter unhealthy behaviors at home, school, or in other settings, I'm able to challenge them.**

	☐Never ☐Seldom ☐Sometimes ☐Usually ☐Always
**30.**	**If my parents or family prepare unhealthy snacks (e.g., chips, fruit roll-ups, corn snacks, etc.) for me to take to school, I accept them.**<br>☐Never ☐Seldom ☐Sometimes ☐Usually ☐Always
**31.**	**If may family were overweight and eating a high fat diet, I would tell them to change their eating habits.**<br>☐Never ☐Seldom ☐Sometimes ☐Usually ☐Always
**32.**	**When I go shopping with my mother or father, I buy healthy snacks such as nuts, raisins, and dried chickpeas instead of chips, snacks, chocolate, and sweets.**<br>☐Never ☐Seldom ☐Sometimes ☐Usually ☐Always
**33.**	**When I go shopping with my mother or father, I buy foods that are certified as healthy.**<br>☐Never ☐Seldom ☐Sometimes ☐Usually ☐Always
**34.**	**When I go shopping with my mother or father, I buy foods with standardized labeling.**<br>☐Never ☐Seldom ☐Sometimes ☐Usually ☐Always
**35.**	**When I go shopping with my mother or father, I buy foods that are not expired.**<br>☐Never ☐Seldom ☐Sometimes ☐Usually ☐Always
**36.**	**When I go shopping with my mother or father, I buy foods with sustainable packaging.**<br>☐Never ☐Seldom ☐Sometimes ☐Usually ☐Always
**37.**	**When I go shopping with my mother or father, I buy foods that are stored appropriately or kept refrigerated.**<br>☐Never ☐Seldom ☐Sometimes ☐Usually ☐Always
**38.**	**I eat food from all the food groups every day.**<br>☐Never ☐Seldom ☐Sometimes ☐Usually ☐Always
**39.**	**I usually try new foods that I've never eaten.**<br>☐Never ☐Seldom ☐Sometimes ☐Usually ☐Always
**40.**	**I usually try new vegetables that I've never eaten.**<br>☐Never ☐Seldom ☐Sometimes ☐Usually ☐Always
**41.**	**I can buy healthy food from the school cafeteria, depending on my pocket money.**<br>☐Never ☐Seldom ☐Sometimes ☐Usually ☐Always
**42.**	**If the school cafeteria doesn't offer any healthy foods, it will be difficult for me to choose healthy snack.**<br>☐Definitely won't ☐Probably won't ☐Maybe ☐Probably will ☐Definitely will

43. Have you ever seen a fact sheet on a food package?

◯ **Yes** ◯ **No**

44. For the following pair of food fact sheets, choose the one you think is more nutritious ("better for you") by filling in the circle next to your choice. Then explain the reasons for your choice.

Nutrition Facts<br>Servings Per Container: 4<br>Serving size: 230gr		
Amount Per Serving<br>Calories: 87.4 Kcal		
Nutrition Facts		%Nutrition Reference Values
Total Fat	2.78 gr	**
Total Carbohydrate	8.51 gr	**
Sugar	0.27 gr	**
Protein	7.13 gr	14.26%
Limit these nutrients		
Saturated Fatty Acid	1.84 gr	9.2%
Trans Fatty Acid	0 gr	**
Sodium	242.65 mg	12.13%
*Nutrition Reference Values not established		

Nutrition Facts		
Calories in 100 gr = 52.92kcal		
Calories from Fat in 100 gr = 28.8kcal		
	Nutrition Facts	% Daily Value In 100 gr
Total Fat	3.2 gr	4.88
Saturated Fatty Acid	2 gr	10
Trans Fatty Acid	0 gr	0
Protein	3.15 gr	4.5
Total Carbohydrate	2.88 gr	1
Sugar	0.36 gr	0.1
Sodium	42 mg	1.75
*Daily Values are based on a 2000 calorie diet		
Total Fat lower than 65gr<br>Saturated Fatty Acid lower than 20gr<br>Sodium lower than 2400mg<br>Carbohydrate lower than 300gr		

Figure B)

◯ **A)** ◯ **B)** **Don't know**

- Why? (Explanation)……

45. **According to the colored information about Tafi (a kind of chocolate), answer the following questions.**

1) **How would you describe the amount of sugar in this product?**
   a) low b) moderate c) high d) don't know

2) **How would you describe the total amount of fat and trans-fatty acid in this product?**
   a) low b) moderate c) high d) don't know

3) **How would you describe the amount of salt in this product?**
   a) low b) moderate c) high d) don't know

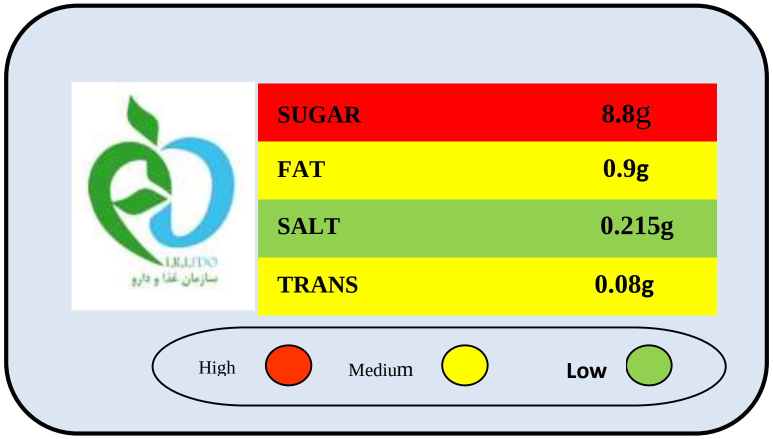

46. **According to the colored information about pasteurized milk, answer the following questions.**

1) **How would you describe the amount of fat in this product?**
   a) low b) moderate c) high d) don't know

2) **How would you describe the amount of salt in this product?**
   a) low b) moderate c) high d) don't know

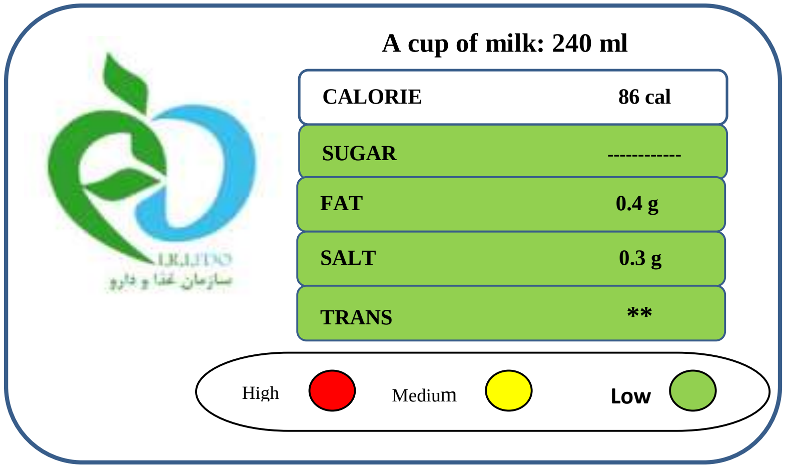

© Doustmohammadian. et al., 2016
